# Epigenetic remodeling by sex hormone receptors and implications for gender affirming hormone therapy

**DOI:** 10.3389/fimmu.2025.1501959

**Published:** 2025-05-08

**Authors:** Den Celestra, Nhi N. L. Nguyen, Camille Laberthonniere, Ken C. Pang, Richard Saffery, Rachel A. Davey, Musa Mhlanga, Ada S. Cheung, Boris Novakovic

**Affiliations:** ^1^ Murdoch Children’s Research Institute and Department of Pediatrics, The University of Melbourne, Parkville, VIC, Australia; ^2^ Radboud Institute for Molecular Life Sciences RIMLS, Radboud University Medical Center, Nijmegen, Netherlands; ^3^ Department of Adolescent Medicine, Royal Children’s Hospital, Parkville, VIC, Australia; ^4^ Department of Medicine Austin Health, University of Melbourne, Heidelberg, VIC, Australia; ^5^ Department of Endocrinology, Austin Health, Melbourne, VIC, Australia

**Keywords:** epigenetics, sex hormones, estrogen, testosterone, estrogen receptor, gender-affirming hormonal therapy, gender, immunity

## Abstract

Sex differences in immune system development and response to pathogens has been well documented, with females exhibiting more favorable outcomes for certain infections but a higher incidence of autoimmune disease compared to males. At least some of these sex differences are mediated by sex hormones, which signal through sex hormone receptors to remodel the regulatory chromatin landscape of cells. Here, we summarize the current knowledge of how sex hormone receptors remodel chromatin structure and epigenetic marks in different contexts in humans. As the epigenome is fundamental to specifying cell identity and function, and reflects past exposures, epigenetic variation can influence cellular responses to future stimuli. This has implications for susceptibility to infection and complex inflammatory disease in a range of hormone therapy settings, including gender-affirming hormone therapy in transgender people. Therefore, profiling of epigenetic marks in the context of gender-affirming hormone therapy is an important unexplored field of research.

## Introduction

Sexual dimorphism describes differences between males and females across various factors, including but not limited to behavior and immunity (1). Sex hormones are one contributor to this dimorphism, where males have higher testosterone and lower estrogen levels, while females have higher estrogen and lower testosterone levels, with age being a major factor in this ratio (2). Sex hormone signaling via sex hormone receptors is a major transcription pathway that influences cellular function, namely cytokine production, cell proliferation, and reactivity (3).

Sex differences in immune function between males and females are also influenced by genetics, with several key genes involved in immunity expressed on the X chromosome. This includes receptors such as TLR7, TLR8, and ACE2 (4, 5), as well as FOXP3, which controls regulatory T cell production (6). Further, the X-chromosome encodes 10% of all miRNAs, including miRNA-18 and 19 which are associated with sex-biased immune response (7, 8).

Functional programming of innate and adaptive immune cells depends on epigenetic remodeling, which alters the regulatory landscape of the genome and controls gene expression (9, 10). These changes influence immune cell identity and can predict how the cell will respond to exogenous stimuli (11). For example, T-cell exhaustion has been linked to epigenetic reprogramming that leads to changes in differentiation trajectory (12, 13). Autoimmune disease is associated with altered cytokine production and immune cell reactivity due to epigenetic changes that are caused by environmental influences, genetic variants, and medication (14). Additionally, certain infections, including SARS-CoV-2 and malaria, as well as immunizations, such as influenza and BCG vaccines, induce changes in the epigenomes of hematopoietic stem cells and monocytes (15–18).

This mini review will summarize recent data on sex hormone receptor-mediated epigenetic remodeling and immune function modulation, with implications for understanding immune function changes in transgender individuals following gender affirming hormone therapy (GAHT). Although not covered in this mini-review, the sex hormone progesterone also plays a vital role in immunity, particularly the promotion of maternal-fetal tolerance during pregnancy, through expanding regulatory T cells and regulation of reactivity of other immune cells (19).

## Sex hormone receptor signaling

Sex hormones influence various physiological systems including neurological, reproductive, musculoskeletal, and immune systems (20, 21). Testosterone, which is a type of androgen, is associated with masculinizing effects, spermatogenesis, and is a modulator of immune response (7, 22). Estrogen is dominantly expressed in females, is associated with feminizing effects, and is a driver of several diseases such as cancer, and a promoter of immune function (7, 23).

### Estrogen receptor

Estrogen receptors (ERs) -α and -ß are nuclear receptors encoded by the *ESR1* and *ESR*2 genes, respectively (24). These receptors are transcriptional regulators that can activate or repress specific genes upon binding of a ligand, leading to changes in chromatin interactions (25, 26). Genomic signaling leads to a conformational change followed by the induction of receptor dimerization wherein the binding site affinity, and specificity of the receptor increase wherein the signaling ultimately influences the change of the gene expression profile (27). In contrast, non-genomic signaling can trigger multiple pathways, such as protein-kinase activation or phosphorylation of transcription factors and activate nuclear ERs to bind to the DNA (28, 29). In addition to binding regulatory elements in DNA, ER-α can also induce posttranslational modifications of proteins upon binding of specific ligands (30).

### Androgen receptor

Androgen receptor (AR) is a nuclear receptor that is encoded by the *AR* gene on the X chromosome (31). Genomic AR signaling mediates transcriptional activity whereby the androgen-AR complex translocates to the nucleus, dimerizes, and binds to androgen-responsive elements (22) to enhance or repress nearby genes (32). Genomic signaling by AR is influenced by coregulators, which can enhance or inhibit transcriptional activity by facilitating chromatin remodeling and histone modifications (33). The non-genomic AR signaling pathway activates intracellular kinase cascades that benefit cell proliferation and survival through targeting plasma membrane proteins or receptors and can also activate phosphorylation pathways (34).

## Epigenetics

Epigenetics literally means ‘above DNA’ and refers to the study of molecular interactions that influence DNA structure, compaction, and function (35). Epigenetic marks can be ‘written’, ‘erased’, and ‘read’ by specific nuclear proteins to regulate gene expression in a range of physiological processes (36, 37). Three major epigenetic modifications are (i) DNA methylation, where a methyl (-CH_3_) group is added to the cytosine nucleotide within a cytosine-guanine sequence (CpG dinucleotide) (38–40); (ii) histone post-translational modifications, such as acetylation and methylation (41); and (iii) non-coding RNAs, which are transcribed RNAs that are not translated into proteins, but can regulate gene expression through mediation of chromatin structure (42).

Most of the genome (over 95%) does not code for proteins. Much of this noncoding landscape plays an important role in regulating the transcription of coding genes (43). Indeed, hormone receptors such as *ESR1* bind large swaths of the noncoding genome, influencing the activity of coding genes (44).

### Epigenetic remodeling by estrogen receptors

Changes in gene expression due to epigenetic remodeling *via* sex hormone receptors is observed in various diseases and physiological processes (**Table 1**). Both ER -α and -ß are expressed in more than 70% of all breast cancers, with estrogen signaling being a driver of carcinogenesis (63). Therefore, much of what we know about how the ER remodels chromatin is based on cancer studies (**Table 1**). These studies illustrate that sex hormone receptors can alter the 3D chromatin landscape, DNA methylation and histone post-translational modifications, by interacting with different chromatin modifiers (**Table 1**).

**Table 1:** Studies on sex hormone receptors and epigenetic remodeling.

Sex Hormone receptor	Context	Finding	Reference
Estrogen Receptor	Breast cancer	Hypermethylation of ER binding sites in Endocrine resistant breast cancer (ER+) cells leads to loss of ER binding to chromatin and loss of 3D chromatin interactions.	(45)
Estrogen Receptor	Breast cancer	Serine starvation depletes H3K27ac at specific ER pathway genes, including the promoter region of *ESR1*.	(46)
Estrogen Receptor	Breast cancer	Mutations in FOXA1 induce a stronger estrogen response through increased binding at ER binding sites, associated with lower response to aromatase inhibitors.	(47)
Estrogen Receptor	Breast cancer	ARID1A regulated luminal cell identity in breast cancer and endocrine therapy response, by regulating genome-wide ER–FOXA1 chromatin interactions and ER-dependent transcription.	(48)
Estrogen Receptor	Breast cancer	The histone demethylase, KDM5, plays a role in cellular heterogeneity in therapeutic resistance. Inhibition of KDM5 increases sensitivity to anti-estrogens by modulating ER signaling and decreasing cellular transcriptomic heterogeneity.	(49)
Estrogen Receptor	Breast development	DNA methylation at the *ESR1* is associated with estrogen response and breast composition in adolescent females that may influence breast cancer risk in adulthood.	(50)
Estrogen Receptor	Breast cancer	Interaction between MAF, ERα, and the histone demethylase, KDM1A, remodels chromatin to promote metastasis.	(51)
Estrogen Receptor	Chronic prostasis/pelvic pain syndrome	*ESR1* and *ESR2* in ejaculated somatic cells of CP/CPPS are hypomethylated and associated with an increase in estradiol levels in seminal plasma. Altered response of cytokine and chemokine expression after estradiol treatment of human mast cell lines was observed.	(52)
Estrogen Receptor	Reproduction	ERα/ß agonist affects spermatogenesis by histone modifications.	(53)
Estrogen Receptor	Endometriosis	Estradiol and P opens chromatin in endometrial stromal fibroblast that affects pathogenicity of endometriosis.	(54)
Estrogen Receptor	Hypertrophic cardiomyopathy	Altered DNA methylation profile was observed in hypertrophic cardiomyopathy which affected altered genes such as ITLN1 related to immune function where *ESR1* gene is at its node.	(55)
Estrogen Receptor	Immune Function	The highly dynamic hormone of female in different timepoints is associated to specific epigenetic changes that contributes to sex-specific differences in immune-mediated and endocrine disease.	(56)
Estrogen Receptor	Spermatogenesis	NH4Cl and/or Na2S disruption of spermatogenesis across generations may involve ERα-regulated changes in DNA and histone methylation.	(57)
Estrogen Receptor	Breast cancer	LATS inhibitor targeting the Hippo pathway suppresses *ESR1* and the growth of ER+ breast cancer cells and tumor organoids through epigenetic changes.	(58)
Estrogen Receptor	Breast cancer	Inhibition of PI3Kα is involved in the opening of chromatin at the ER target loci and enhances KMT2D activity. The phosphorylation of KMT2D attenuates methyltransferase activity and ER function.	(59)
Estrogen Receptor	Chromatin interactions	Chromatin interactions by extensive chromatin looping is utilized by ERα for transcription regulation.	(26)
Estrogen Receptor	Gender-affirming hormone therapy	DNA methylation of region III (RIII) of the *ESR1* promoter is altered by both feminizing and masculinizing GAHT.	(60)
Androgen Receptor	Prostate Cancer	AR and MYC gene loci show similarities wherein both were androgen-repressed by chromatin changes.	(61)
Androgen Receptor	Prostate Cancer	Androgen receptor enhancers are extremely heterogeneous and associated with chromatin remodeling that impacts prostate cancer susceptibility to treatment.	(62)

P, progesterone.

Epigenetic remodeling by ERs also plays a role in non-cancer settings, such as breast development, where the DNA methylation signature of adolescent girls is dependent on estrogen response and breast composition, which may influence breast cancer risk in adulthood (50). Hormonal changes in females and males during puberty were found to influence DNA methylation near predicted estrogen-responsive genes (56). In addition, disruption of DNA methylation at the *ESR1* gene locus by endocrine-disrupting chemicals has been associated with decreased male fertility due to the decline of sperm quality (53, 57).

### Epigenetic remodeling by androgen receptor

AR can also remodel chromatin, but these effects are much less studied than for ER. Most studies looked at the role of AR in prostate cancer, where the AR drives epigenetic heterogeneity at enhancers through AR binding sites, affecting response to therapy (62). There is a significant knowledge gap in our understanding of how these AR-mediated changes within the epigenome shape cellular function in other tissues and outside the prostate.

## Sex differences in immunology

Sex differences are observed in immune responses to infections, with females generally exhibiting hyper reactivity than males (7). This is particularly evident in influenza infections and following influenza vaccination, where females produce higher neutralizing antibodies and inflammatory cytokines (64–66). Post-influenza vaccination, females show elevated levels of inflammatory markers such as leptin, or interleukin-receptor agonist (IL-1RA) (64, 67). Higher circulating estradiol concentrations in females reduce other proinflammatory cytokines like tumor necrosis factor (TNF)-α and chemokine ligand (CCL)-2, primarily mediated via the ERα, thereby lowering influenza-related morbidity and mortality (68). Conversely, males, influenced by the immunosuppressive effects of testosterone, generally exhibit weaker responses to influenza vaccination, with lower antibody production, especially in those with high serum testosterone concentrations (64, 66, 69). These sex differences extend to COVID-19, where males typically experience more severe outcomes, partly due to higher transmembrane protease, serine 2 or TMPRSS2 expression facilitating viral entry (70). Conversely, the effects of estradiol on angiotensin-converting enzyme 2 and angiotensin II receptor type 1 signaling in females reduce the severity of COVID-19 infection (71, 72). Males with long COVID cognitive symptoms show higher levels of the neuroinflammation-linked chemokine CCL11 compared to females (73), suggesting increased susceptibility to certain post-COVID neuroinflammatory effects. Together, these findings highlight the complex relationship between sex hormones and immune responses in viral infections.

In autoimmune disease, sexual dimorphism leads to stronger, estrogen-driven immune responses in females, increasing their overall susceptibility (74, 75). For example, in juvenile idiopathic arthritis (JIA), females are more susceptible to chronic inflammation with three to six females for every male patient are affected due to increased activation of immature neutrophil-related genes, leading to enhanced neutrophil activation that may impact treatment effectiveness, including responses to Interleukin (IL)-1 receptor antagonists (76). Similarly, females are more prone to developing multiple sclerosis (MS), with a 3:1 ratio compared to males, partly due to estrogen-enhanced IL-17 expression, which drives autoimmune pathogenesis by boosting pro-inflammatory cytokine production and T cell activity (75, 77, 78). Females also experience more severe skin inflammation, particularly in psoriasis, due to estrogen amplifying the inflammatory response by increasing cytokine production and immune cell activity (79). While genetic differences between males and females are a factor in this dimorphism, most differences are attributable to sex hormones receptors as well (80).

## Sex hormones and immunity

Sex hormone receptor signaling in immune cells influences various functions such as cell proliferation, reactivity, and overall function (81). **Table 2** summarizes studies on how sex hormone signaling affects immune cells.

**Table 2:** Studies on sex hormone receptors and immunology.

Sex hormone receptor	Context/Disease	Immune subtype	Finding	Reference
Estrogen Receptor	Autoimmunity	Follicular T cells (Tfh)	CD4-ERα KO mice resulted to increased autoantibody production and Tfh cells reactivity. Treatment of estradiol in wildtype mice suppressed mRNA expression of Bcl-6 and IL 21.	(82)
Estrogen Receptor	Allergy	Group 2 innate lymphoid cells (ILC2)	ER- α mediates airway inflammation.	(83)
Estrogen Receptor	Chlamydia	Regulatory T cells	Estradiol treated murine models inhibited C. muridarum infection while affecting T cell reactivity and IFNe production.	(84)
Estrogen Receptor	Psoriasis	Neutrophils and Macrophage	Estradiol has a pathogenic role in promoting skin inflammation in psoriasis by affecting both upstream and downstream processes of transcription via ER-ß.	(85)
Estrogen Receptor	Rheumatoid Arthritis	CD4+ T cells	CD2 polymorphisms are linked to rheumatoid arthritis, and estradiol regulation of CD2 in T cells suggests hormonal influence on CD2 contributes to sex differences in autoimmune diseases	(86)
Estrogen Receptor	Colon Inflammation	Macrophage	Intestinal ERβ, activated by TNFα, reduces colon adenomas by inhibiting TNFα/NFκB signaling. ERβ represses NFκB, upregulates inhibitor ATF3, and decreases CCL2 and CCL4 secretion, reducing pro-inflammatory macrophage recruitment.	(87)
Estrogen Receptor	Colitis	T cell	ERα in T cells is a vital receptor for its reactivity and activation affecting Foxp3 expression.	(81)
Not specified	Sex difference	NK, B, and T cell	Higher B and T cell proportion in females but higher NK cell proportion in male.	(88)
Not specified	Infection/Sepsis	Neutrophils	Human male neutrophils produce more TNF, and showed higher responsive towards LPS and IFNγ stimulation	(89)
Estrogen Receptor and Glucocorticoid Receptor	Asthma	T helper 2 (Th2)	Circulating Th2 and type 2 cytokines are significantly higher in females with severe asthma due to synergistic effect of ER-GC.	(90)
Estrogen Receptor	HIV	Th17	ER signaling is responsible for the latency of HIV pathogenesis.	(91)
Androgen Receptor	Influenza	CD8+ T cells, eosinophils	AR inhibits cytokine production, degranulation influenza A virus-specific CD8+ T cells, and eosinophils into the lungs after influenza infection.	(92)
Androgen Receptor	Asthma	Th2 and Th17	AR agonist in mice decrease helper T cells 2 and 17’s reactivity which improved lung function in asthmatic mouse.	(93)
Androgen Receptor	Asthma	Treg	AR signaling inhibited Treg reactivity during asthma challenge in mouse model.	(94)
Androgen Receptor	Renal Inflammation	–	Blocking of AR by an antagonist is associated with a decrease in renal tissue inflammation, fibrosis, and apoptosis via multiple cytokine-mediated pathways.	(95)
Androgen Receptor	Immunotherapy resistance in prostate cancer	CD8+ T cells	AR blockade in CD8+ T cells resulted to PD-1 inhibition and promotion of T cell function and production of IFNy by harboring open chromatin regions.	(96)
Androgen Receptor	Antitumor Immunity	CD8+ T cells	CD8+ T cells exhibit sexual dimorphism in how it perceives antitumor immunity. AR signaling in male inhibited the activity and stemness of infiltrating CD8+ T cells via epigenetic and transcriptomic remodeling.	(97)
Androgen Receptor	HER2+ Breast Cancer	Macrophage 2 (Tumor associated macrophage)	High testosterone level means low immune cell infiltration in HER2+, trastuzumab-treated breast cancer.	(98)
Androgen Receptor	Female Reproduction	Tregs	AR signaling increase *Foxp3* expression in T regulatory cells in females in ovulatory phase of the menstrual cycle, and thus remodel the acetylation profile of histone H4.	(99)
Androgen Receptor	Anti-pathogenic activity	Neutrophil	Testosterone treatment of human neutrophils increased their phagocytic capacity and decreased microbicidal activity.	(100)

### Estrogen receptor and immunity

CD4-ERα knockout (KO) female mouse model showed a mild autoimmune phenotype with increased autoantibody and follicular helper T cells (TFH) production (82), while polymorphisms in ER binding site affect rheumatoid arthritis by introducing a sex bias Cd2 expression to regulate T cell activation (86). Skin inflammation also depends on endogenous estradiol in mice where a psoriasis ER mouse KO increased IL-17A and IL-1ß production (85). In the context of infection, response to *Chlamydia muridarum* and hepatitis B virus (HBV) in mouse models were both influenced by polymorphisms in *ESR1* (84, 101). The role of ERs in inflammation in the colon, liver, and airway is also evident in mouse studies where TNFα activates intestinal Er-β, while a reduction in ERα increases NF-kB activity through the liver receptor homolog (LRH-1) (87, 102). Additionally, ERα plays a role in allergy, elevating IL-33 release and ILC2-mediated airway inflammation upon allergen challenge (83).

In human studies, sex-based differences in immune cell composition are also observed where males have higher proportion of nature killer (NK) cells subsets, while females exhibit greater abundance of B cell subset (88). Male neutrophils also show higher TNF expression after lipopolysaccharide (LPS) stimulation, which is attributed to increased TLR4 expression in males which may influence sepsis response (89). In females with severe asthma, dual activation of ERα and glucocorticoid receptor synergistically enhances the production of circulating T helper (Th2) cells and type 2 cytokines (90). Finally, *ESR1* has been identified to be an integral regulator in HIV-1 infection with females displaying lower inducible HIV-1 RNA reservoirs compared to males (91).

### Androgen receptor and immunity

AR signaling has been reported to exert inhibitory actions in a number of immune responses (103). For example, in mouse models of influenza, testosterone inhibits influenza A virus (IAV) pathogenesis by systematically modulating CD8+ T cell reactivity (92). Similarly, in a mouse model of asthma, treatment with an allergen, *Alterneria* extract, decreases helper T cell reactivity, while the suppressive function of Tregs is promoted (93, 94). AR signaling also plays an important role in the inflammatory response in the kidney and liver. Treatment of male rats with flutamide, an AR antagonist, has been reported to systematically downregulate cytokines in renal fibrosis, while an increased AR expression was observed in mice during severe infection with HBV (95, 104). Sex-specific differences in anti-tumor immune responses in mice have been attributed, at least in part, to AR-mediated epigenetic remodeling of CD8+ T cells leading to lower reactivity and stemness in the tumor environment (97).

In human studies, testosterone reduces oxidative stress in neutrophils, increasing their phagocytic capacity while decreasing their microbicidal activity (100). AR signaling also increased *Foxp3* expression in regulatory T cells of females during the ovulatory phase of the menstrual cycle, leading to changes in the acetylation profile of histone H4 (99). The importance of AR signaling is further highlighted by a study that showed that inhibition of AR activity in CD8+ T cells prevented T cell exhaustion and improved responsiveness to PD-1 targeted therapy (96). Finally, AR expression inversely correlated with the production of M2 tumor-associated macrophage, CD3+, and CD8+ T cell infiltration in trastuzumab-treated HER 2-positive breast cancer patients, suggesting a stronger role of AR in immune cells within cancer metastasis and proliferation (98).

This section mainly reviewed previous animal studies due to the scarcity of information on how sex hormone signaling affects functional human immune response in various contexts such as autoimmunity and other diseases. Several previous reviews have elucidated the role of sex hormone signaling in immune cells (7, 103, 105) but it is still unclear on how this signaling pathway affects immunity and mechanistically affects diseases associated with it. Overall, these studies highlight that both the ER and AR regulate a wide range of immune cell functions, explaining the sexual dimorphism in inflammation, cancer response, and disease susceptibility.

### Role of epigenetic landscape in immune sex-differences

Epigenetic remodeling at promoters and distal regulatory elements is a key mechanism by which transcription factors establish immune lineages from hematopoietic stem cells (106). Further, exogenous stimuli such as microbial compounds and certain vaccines can induce new regulatory elements in differentiated immune cells, such as monocytes, through changes in histone modifications (107), or DNA methylation (108). Likewise, the tissue microenvironment shapes the epigenetic landscape in tissue resident macrophages (109) and T cells (110). As noted previously, several regulators of immune cell identity and function are located on the X chromosome. For example, *in vitro*-activated B cell subsets from adult and pediatric systemic lupus erythematosus (SLE) patients were found to exhibit disrupted X-chromosome inactivation (XCI), which is speculated to result from an increase in inflammatory cytokines and type I interferons (111). Further, UTX, a master epigenetic regulator that escapes XCI in both human and mouse models was found to control chromatin accessibility and gene expression patterns in NK cells (112). Therefore, sex-differences in immune cell phenotype and function can arise through epigenetic errors in X chromosome inactivation or epigenetic remodeling by sex hormone receptors.

## Implications for gender affirming hormone therapy

At least 1.5 million people in the United States are transgender (~0.5%), of whom 90% have considered or are undergoing GAHT (113), with transgender women having a disproportionately higher rate of HIV infection (114). A cross-sectional study comparing transgender women and men receiving GAHT to cis-men and women, revealed that GAHT influences both the proportions in the circulation and the transcriptome of regulatory T (Treg) cells (115). Interestingly, sex hormones and chromosomes may collectively influence some cell proportions. Peckham et al. found that CD19+ CD27+ IgD- classical-switched memory B cells were sensitive to estrogen only in the XX karyotype, decreasing in transgender women following GAHT, but in post-menopausal cis-women following HRT, but not influenced by estrogen GAHT in transgender women (116). Collectively, these findings show that GAHT leads to a unique impact on different immune cell subtypes. The long-term effect of GAHT on the immune system has not been extensively studied which has clinical implications given the known sex-specific prevalence and risks in many inflammatory, infective and autoimmune diseases. To address this significant knowledge gap, we have longitudinally profiled the immune system of transgender women and transgender men newly commencing GAHT. A significant advantage to this approach is it allows us to dissect the contribution of sex hormone action relative to sex chromosomes in immune function, which is usually difficult to separate from the underlying genetic differences between males and females. Using longitudinal GAHT cohorts, we have shown that this therapy can influence epigenetic marks in blood cells affecting the immune response. A notable example of this is differentially methylated CpG site in the promoter of *IL-21*, which gained DNA methylation after 12 months of masculinizing GAHT, but lost DNA methylation after 12 months of feminizing GAHT (117). We further elucidated the effects of feminizing GAHT in the metabolome wherein a cyproterone-acetate specific decrease in glutamine levels, an important amino acid related to immune cell metabolism, has been observed (118). Altogether, these findings suggest that GAHT influences the changes within the immune cell in a transcriptional level. Recently, Lakshmikanth et al., showed that masculinizing GAHT increased chromatin accessibility at canonical NFkB binding sites in T and NK cells after 12 months of therapy together with the promotion of monocyte responsiveness, together with downstream upregulation of NF-kB and interferon-γ production in NK cells (119). Modulation of interferon signaling by masculinizing GAHT was previously reported, with a decline in IFN-I production by plasmacytoid dendritic cells through the regulation of TLR7/8 (120). This testosterone-associated reduction in IFN-I responses was in line with lower TLR7 responses in males compared to females (121). Therefore, approaches that study immune cell phenotypes, including membrane and nuclear receptors, and downstream chromatin remodeling will provide insights into how sex hormones signal in primary human cells, and potentially explain the contribution of sex hormones to sexual dimorphism in inflammation and development of complex immune diseases.

## Conclusion

Epigenetic remodeling through sex hormone signaling may have wide-ranging impacts on the immune profile of transgender men and women. We highlight that most of our knowledge about how sex hormone receptors remodel chromatin come from cancer studies, and that human studies in the context of hormone change are warranted. Considering that AR and ERs are expressed in a range of tissues and immune cell types, changes in circulating sex hormone concentrations can influence cell responses in the circulation, as well as progenitor populations in the bone marrow. Based on the literature we presented, estrogen and testosterone do not simply promote or inhibit immune responses, but changes in their concentrations would lead to a unique immune cell phenotype in the context of GAHT. Profiling the effects of GAHT on epigenetic remodeling will not only provide insight into the role of sex hormones in immune function and the development of complex immune diseases, but will help inform the healthcare of transgender people on GAHT.
